# The global research and emerging trends in autophagy of pancreatic cancer: A bibliometric and visualized study

**DOI:** 10.3389/fonc.2022.987026

**Published:** 2022-10-03

**Authors:** Mingyang Song, Qin Lu, Min Xu, Yajie Li, Yawen Zhao, Chen Gong, Xilong Ou

**Affiliations:** ^1^ Department of Gastroenterology, Zhongda Hospital, School of Medicine, Southeast University, Nanjing, China; ^2^ Department of Human Anatomy, School of Medicine, Southeast University, Nanjing, China; ^3^ Department of Gerontology, Zhongda Hospital, School of Medicine, Southeast University, Nanjing, China; ^4^ Department of Gastroenterology, Taicang Affiliated Hospital of Soochow University, The First People’s Hospital of Taicang, Jiangsu, China

**Keywords:** pancreatic cancer, autophagy, bibliometric study, apoptosis, gemcitabine, chemotherapy resistance

## Abstract

**Objective:**

To present the global research features and hotspots, and forecast the emerging trends by conducting a bibliometric analysis based on literature related to autophagy of pancreatic cancer from 2011 to 2022.

**Methods:**

The literature data regarding autophagy of pancreatic cancer were retrieved and downloaded from the Web of Science Core Collection (WOSCC) from Clarivate Analytics on June 10th, 2022. VOSviewer (version 1.6.18) was used to perform the bibliometric analysis.

**Results:**

A total of 616 studies written by 3993 authors, covered 45 countries and 871 organizations, published in 263 journals and co-cited 28152 references from 2719 journals. China (n=260, 42.2%) and the United States (n=211, 34.3%) were the most frequent publishers and collaborated closely. However, publications from China had a low average number of citations (25.35 times per paper). The output of University of Texas MD Anderson Cancer Center ranked the first with 26 papers (accounting for 4.2% of the total publications). *Cancers* (n=23, 3.7%; Impact Factor = 6.639) published most papers in this field and was very pleasure to accept related researches. Daolin Tang and Rui Kang published the most papers (n=18, respectively). The research hotspots mainly focused on the mechanisms of autophagy in tumor onset and progression, the role of autophagy in tumor apoptosis, and autophagy-related drugs in treating pancreatic cancer (especially combined therapy). The emerging topics were chemotherapy resistance mediated by autophagy, tumor microenvironment related to autophagy, autophagy-depended epithelial-mesenchymal transition (EMT), mitophagy, and the role of autophagy in tumor invasion.

**Conclusion:**

Attention has been increasing in autophagy of pancreatic cancer over the past 12 years. Our results undoubtedly provide scholars with new clues and ideas in this field.

## Introduction

Pancreatic cancer remains the most aggressive and fatal among all malignancies, with a dismal 5-year relative survival rates of only 11%. Approximately 62,210 new pancreatic cancer cases are expected in the US in 2022 (1). Pancreatic ductal adenocarcinoma (PDAC) is the majority (90%) of pancreatic cancers. Most patients with pancreatic cancer are not suitable for curative surgery because of an advanced or metastatic stage at the time of diagnosis (2). Over the past decade, even the most advanced diagnostic tools, perioperative management, and systemic anti-tumor therapy for advanced disease have been developed but only modest improvements in patient outcomes (3). Therefore, early diagnosis, mechanisms of tumorigenesis, and anti-tumor strategies of pancreatic cancer have always been research hotspots.

Autophagy is an evolutionarily conserved catabolic mechanism that damaged organelles, aggregated proteins, cytoplasmic macromolecules, or pathogen are delivered to lysosomes for degradation, providing macromolecular precursors and energy, and ultimately recycled back into the cytosol for reuse (4–12). Based on diverse cellular functions, autophagy broadly encompasses three types: macroautophagy, microautophagy, and chaperone-mediated autophagy (13). Macroautophagy is the main autophagy process (hereafter autophagy) in which the autophagosome is newly formed by a double-membrane vesicle to sequester a variety of cellular cargo and transport this autophagic material to lysosomes for subsequent degradation (14, 15). Autophagy can be selective and non-selective depending on the way of sequestration of degradation targets. Non-selective autophagy is responsible for randomly engulfing cytoplasmic components into phagophores (the precursors to autophagosomes), whereas selective autophagy identifies and removes specific components. Selective autophagic degradation processes include mitophagy for damaged and/or superfluous mitochondria, aggrephagy for protein aggregates, ferritinophagy for the iron-sequestering protein ferritin, xenophagy for intracellular pathogens and the like (16–19). By contrast, microautophagy is responsible for directly engulfing cellular cargo by lysosomes (20). Finally, chaperone-mediated autophagy involves the direct translocation of specific cytosolic proteins (and possibly DNA and RNA) across the lysosomal membrane with the assistance of HSC70 and other co-chaperones (21, 22). In virtually all eukaryotic cells, autophagy occurs at a low basal level in physiological condition to maintain cellular homeostasis or regulate cellular functions (23, 24). Given the catabolic degradation function of autophagy, it is not surprising that dysregulation of autophagy has been associated with numerous human diseases, including cancers, neurodegenerative diseases, autoimmune disorders, and inflammatory diseases (25, 26). A total of 18,881 autophagy-related articles were published before 2019 and relevant research has dramatically risen in the past decade (27). Among which, the relationship between autophagy and cancer is one of the research hotspots. In 2011, Yang et al. reported that pancreatic cancers have a distinct dependence on autophagy (28). Then, hundreds of research articles have been published on autophagy of pancreatic cancer. Thus, it is urgently needed to collect and analyze the vast quantities of literatures on this topic.

Bibliometrics is a quantitative science based on large volumes of literatures. It can use of mathematical and statistical methods to comprehensively analyze the authors, keywords, journals, countries, institutions, citations, and their associations of selected publications, thus providing an objective evaluation of the dynamics and emerging trends in a research field or discipline (29). The visualization of bibliometric analysis can demonstrate the results in different forms and contribute to data interpretation, which make the results more intuitive and comprehensive (29, 30). This method has been widely used to assess various research domains, including medicine (31–33).

Previous bibliometric studies has focused on the research of autophagy (27), mitophagy (34), pancreatic cancer (35), tumor microenvironment of pancreatic cancer (36), and pancreatic neuroendocrine tumors (37). As a novel perspective, we conducted a bibliometric analysis based on literature related to autophagy of pancreatic cancer from 2011 to 2022. This study aims to present the global research features and hotspots, and forecast the emerging trends in that field, which may provide researchers with new clues and ideas in the field of autophagy and pancreatic cancer.

## Materials and methods

### Data screening and collection

Web of Science Core Collection (WOSCC) is the most frequently used database in bibliometric analysis (31). We retrieved and downloaded literature data in the WOSCC from Clarivate Analytics on June 10th, 2022. Primary search terms were “pancreatic cancer”, “pancreatic carcinoma”, “pancreatic ductal adenocarcinoma” and “autophagy” and detailed search strategy is provided in **Supplemental File S1**. The retrieval time was set from January 1st, 2011 to June 9th, 2022. The language was limited to English and the literature type we searched for was restricted to article or review article. Two authors (MY S and Q L) independently screened the search results and removed the paper that did not related to autophagy of pancreatic cancer by reading the title, abstract, and if necessary, the whole article. Different viewpoints would be resolved by negotiation or reviewed by an experienced corresponding author (XL O). The literature data was finally exported with the record content of “Full Record and Cited References” and downloaded in plain text format.

### Data analysis and visualization

We used VOSviewer (version 1.6.18) to perform the bibliometric analysis based on the literature data. The annual output of publications related to autophagy of pancreatic cancer was plotted using GraphPad Prism (version 6.0.4).

**Figure 1:**
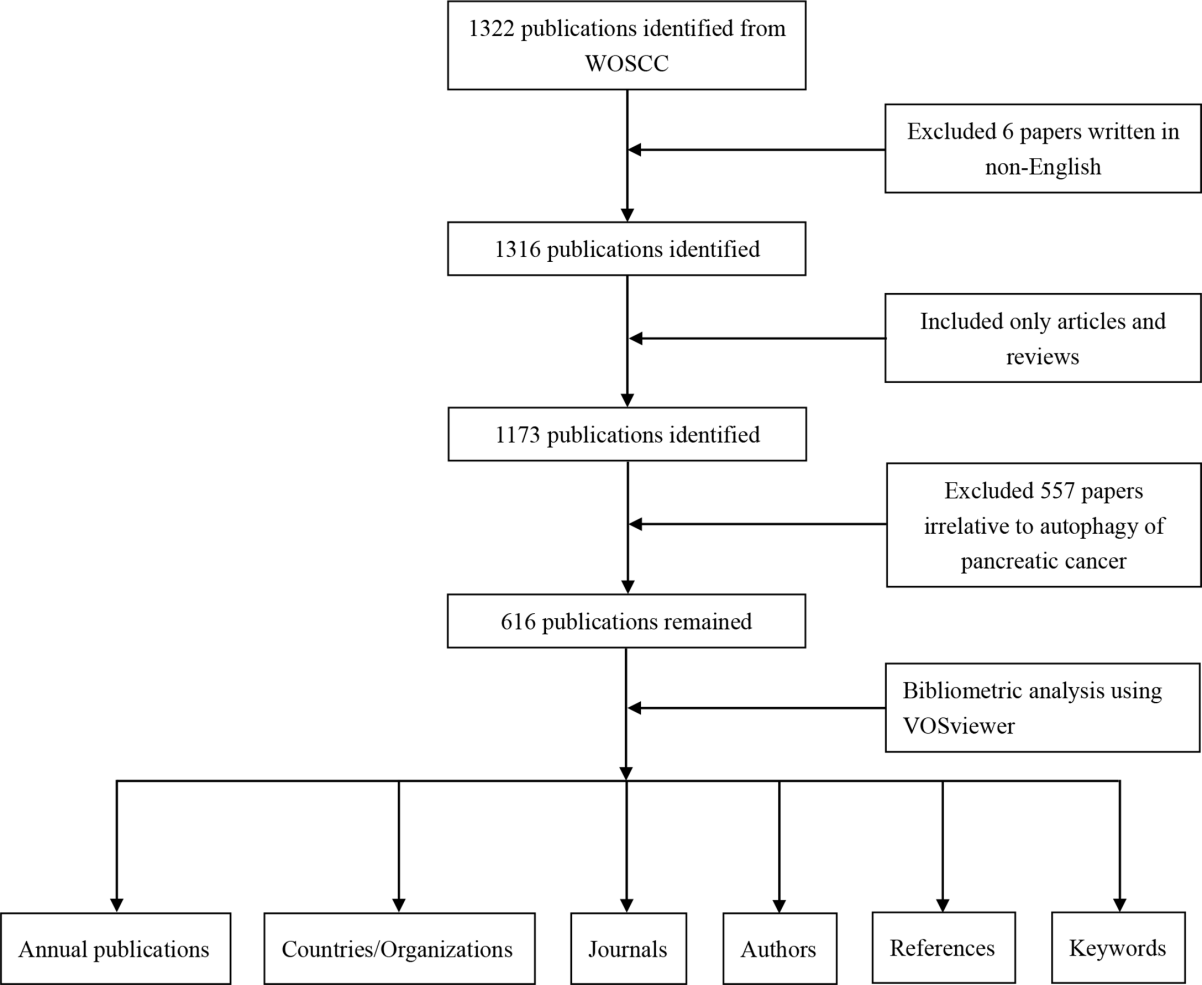
Flow chart of the data collection and analysis for research on autophagy of pancreatic cancer.

VOSviewer is a free JAVA-based computer program developed by Van Eck and Waltman, which is used for constructing and generating bibliometric maps visually. It provides a variety of easy-to-interpret visualization maps, including network visualization, overlay visualization, and density visualization (38). In VOSviewer, the co-authorship network map of countries/organizations/authors, the overlay visualization map of the citation analysis of sources, the density map of the co-citation analysis of cited authors and the co-occurrence analysis of all keywords were built. The data analyzing flow chart can be seen from **Figure 1**.

## Results

### Publication outputs and trend

**Figure 2:**
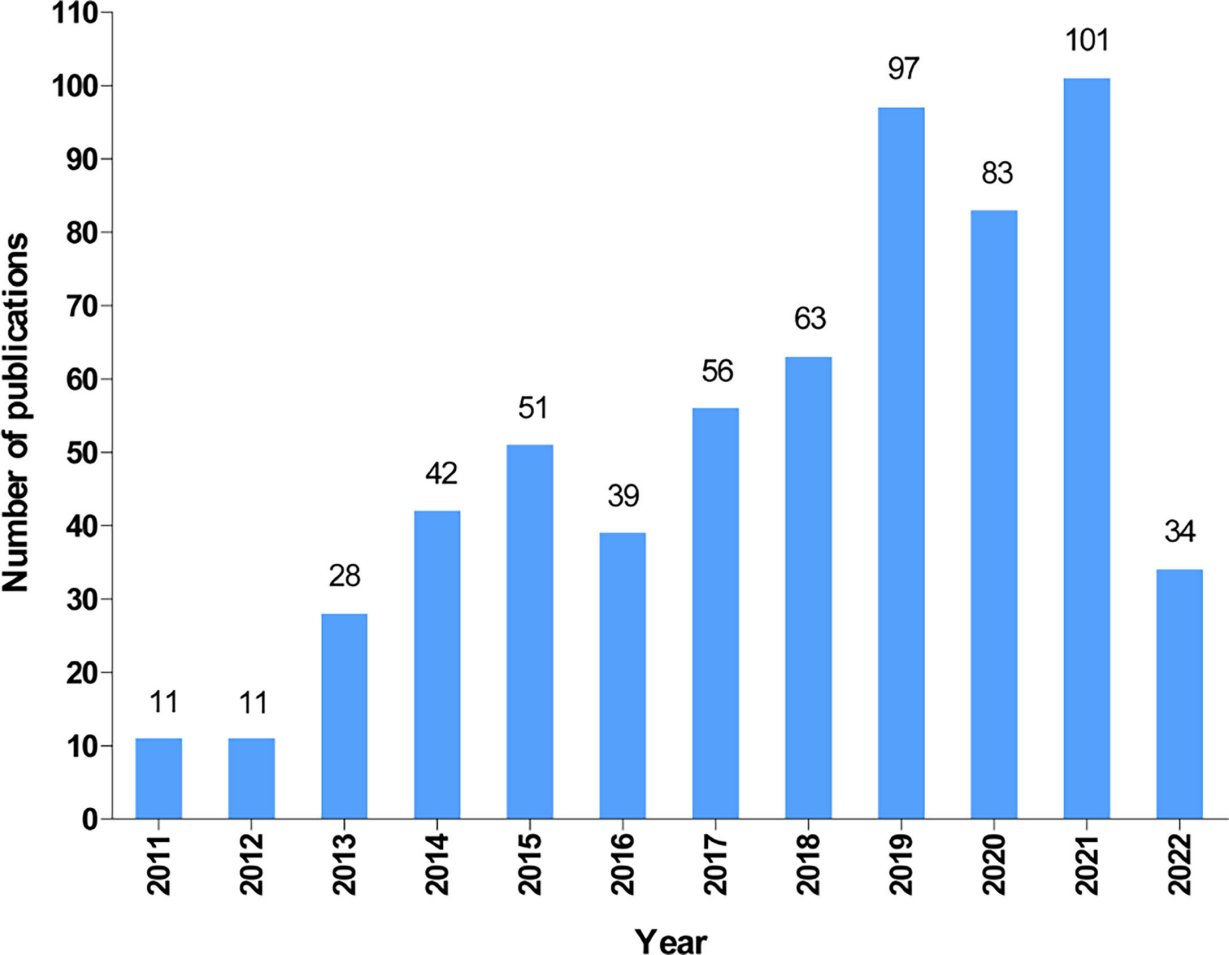
The annual output of autophagy and pancreatic cancer from 2011 to 2022.

According to our search strategy, a total of 616 publications on autophagy of pancreatic cancer were remained for bibliometric analysis, including 479 articles (77.8%) and 137 reviews (22.2%). The annual number of publications on the autophagy of pancreatic cancer from 2011 to 2022 (June 9th, 2022) is presented in **Figure 2**. Generally, the number of publications increased year by year and it dramatically raised from 11 in 2011 to 101 (including 66 articles and 35 reviews) in 2021, suggesting a gradually increased attention to autophagy of pancreatic cancer over the past 12 years. Moreover, as of June 9th, 2022, a total of 34 papers (including 31 articles and 3 reviews) have been published.

### Countries and organizations

**Figure 3:**
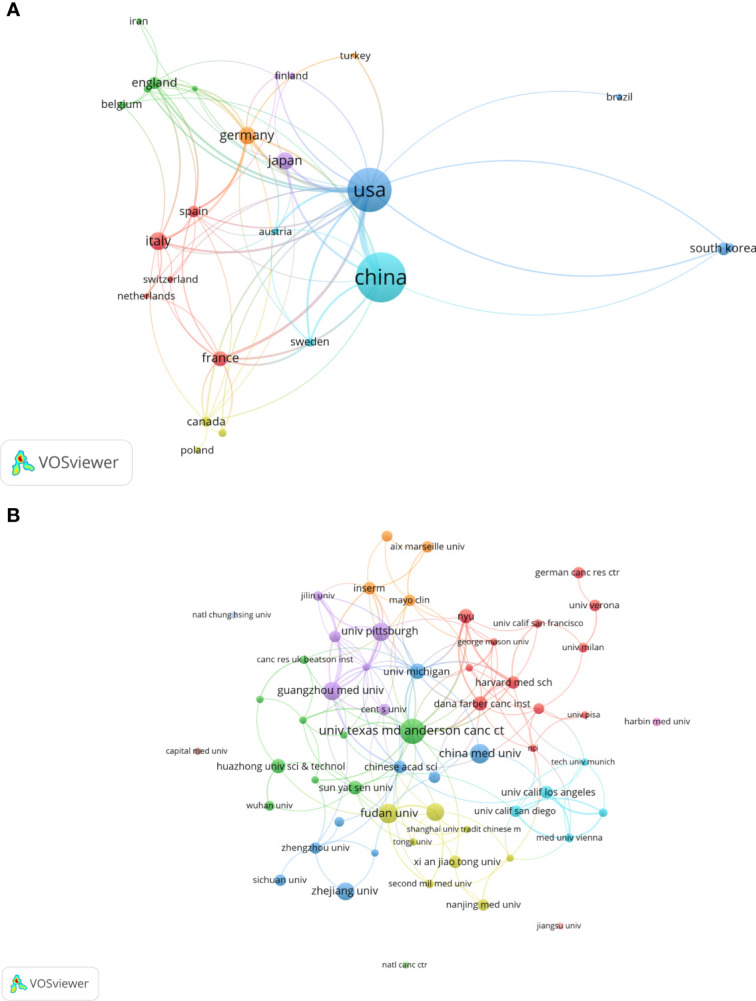
A visual map for VOSviewer network of countries and organizations related to autophagy of pancreatic cancer. **(A)** Country cooperation analysis. The total link strength was 241; the layout parameters: Attraction: 2, Repulsion: 1; **(B)** Organization cooperation analysis. The total link strength was 266; the layout parameters: Attraction: 4, Repulsion: -1. The circle size means the number of publications; the thickness of the line means the strength of the connection.

All included publications in the field covered 45 countries and 871 organizations. The output of China ranked the first with 260 (accounting for 42.2% of the total publications), followed by the United States (n=211, 34.3%), Italy (n=40, 6.5%), Germany (n=37, 6.0%) and Japan (n=37, 6.0%) (**Table 1**). However, among the top 10 countries, publications from China had a low average number of citations (25.35 times per paper), while the United States (63.9 times) was in first place by the average number of citations, followed by Italy (62.4 times), Germany (49.08 times), England (39.95 times) and France (39.18 times). Besides, a co-authorship network map of countries was built (**Figure 3A**) as the cooperation between different countries can be considered as a measure of international cooperation. Only the countries with a minimum of five publications were included and 25 countries were subsequently identified. China collaborated closely with the United States. The United States cooperated with 23 countries, ranked first, followed by Germany (n=12), Spain (n=11), China (n=10), Italy (n=10), and England (n=10).

**Table 1:** The top 10 productive countries in the field of autophagy and pancreatic cancer.

Rank	Country	Publications	Percentage	Total citations	Average citation
1	China	260	42.2%	6592	25.35
2	United States	211	34.3%	13482	63.9
3	Italy	40	6.5%	2456	62.4
4	Germany	37	6.0%	1816	49.08
5	Japan	37	6.0%	1069	28.89
6	France	28	4.5%	1097	39.18
7	South Korea	22	3.6%	526	23.91
8	England	19	3.1%	759	39.95
9	Spain	18	2.9%	452	25.11
10	Canada	13	2.1%	340	26.15

The top 11 active organizations based on publication number were listed in **Table 2**. The production from these organizations ranged 12 to 26 publications, accounting for 28.7% (177/616) of the total publications. Organizations from China and the United States account for 6 and 5 respectively. University of Texas MD Anderson Cancer Center contributed the most publications (n=26, 4.2%) with 2657 citations, followed by Fudan University (n=18, 2.9%) with 645 citations and China Medical University (n=18, 2.9%) with 310 citations. Publications from China organizations had also a low average number of citations. Notably, among the top 11 organizations, Dana-Farber Cancer Institute published a relatively low number of 12 papers related to autophagy of pancreatic cancer from 2011 to 2022. But it had the highest average number of citations (169.58 times). Five documents were set as a minimum for each organization to be analyzed; therefore, 58 of 871 organizations were included for network analysis (**Figure 3B**). The cooperation between organizations was a little stronger than that between countries based on the total link strength.

**Table 2:** The top 11 productive organizations published literature related to autophagy of pancreatic cancer.

Rank	Organization	Country	Publications	Total citations	Average citation
1	University of Texas MD Anderson Cancer Center	United States	26	2657	102.19
2	Fudan University	China	18	645	35.83
3	China Medical University	China	18	310	17.22
4	Guangzhou Medical University	China	17	1183	69.59
5	University of Pittsburgh	United States	17	1316	77.41
6	Zhejiang University	China	16	214	13.38
7	Shanghai Jiao Tong University	China	16	218	13.63
8	University of Michigan	United States	13	1683	129.46
9	New York University	United States	12	1621	135.08
10	Dana-Farber Cancer Institute	United States	12	2035	169.58
10	Huazhong University of Science and Technology	China	12	268	22.33

### Analysis of journals and co-cited journals

**Figure 4:**
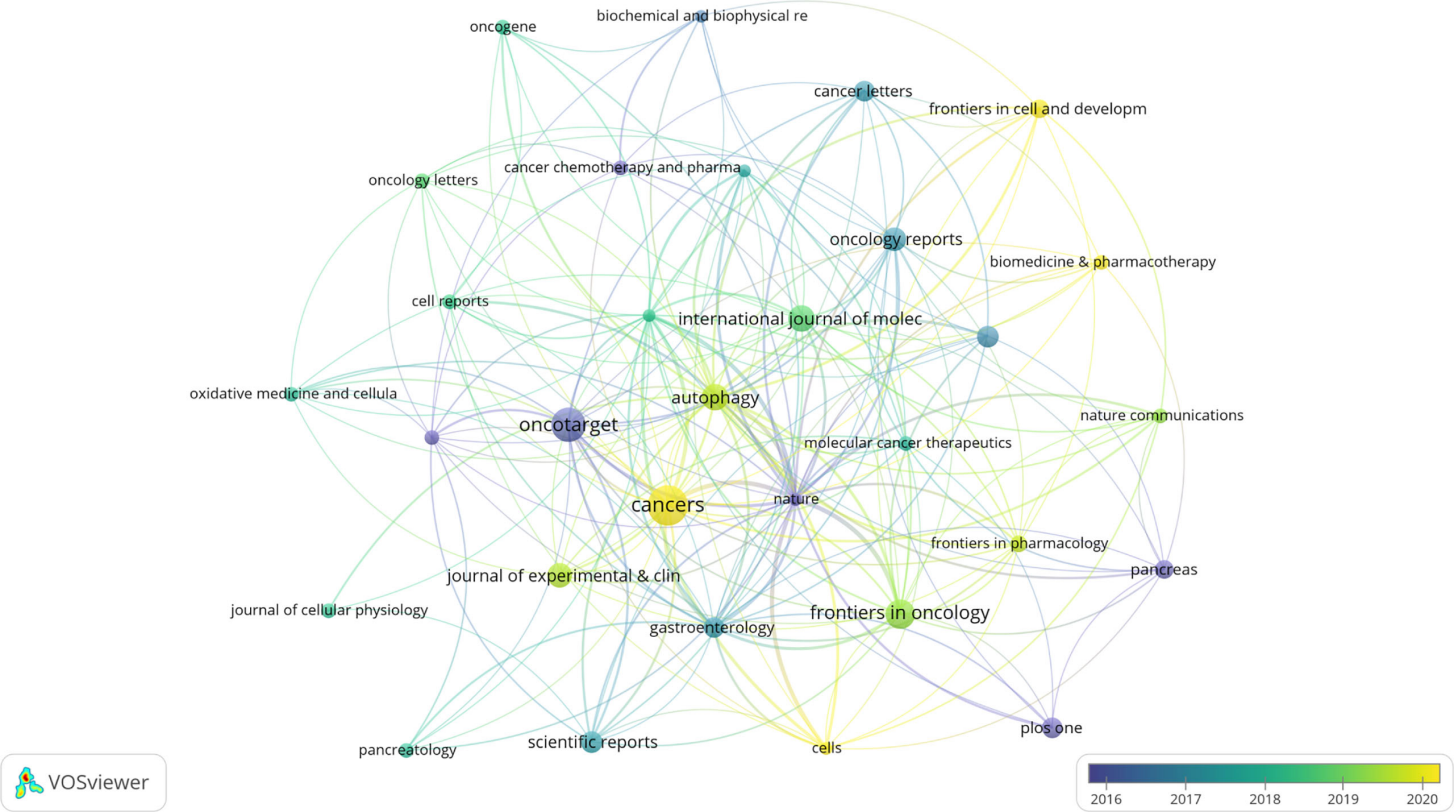
The overlay visualization map of journals associated with research on autophagy of pancreatic cancer. The layout parameters: Attraction: 4, Repulsion: -1. The circle size means the number of publications; the circle colors mean the average published year.

A total of 263 academic journals published the 616 publications on autophagy of pancreatic cancer between 2011 to 2022. As is displayed in **Table 3**, the top 12 most frequent journals were distributed 153 papers, accounting 24.8% for all the obtained publications. The most productive journal has been *Cancers* with 23 papers (3.7% of the total), followed by *Oncotarget* (19, 3.0%), *Frontiers in Oncology* (15, 2.4%), *Autophagy* (13, 2.1%), and *International Journal of Molecular Sciences* (13, 2.1%). The top 3 journals with the highest total number of citations were *Autophagy* (982 citations), *Oncotarget* (841 citations), and *Gastroenterology* (713 citations). Among the top 12 journals, 66.7% (8/12) had an Impact Factor (IF) of more than five, and 66.7% (8/12) were at the Q1 JCR division. Besides, the journals (n=31) published a minimum of five publications were included in the overlay visualization map (**Figure 4**). The yellow nodes represented the emerging journals in recent 3 years. *Cancers* (IF=6.639, Q1), *Frontiers in Cell and Developmental Biology* (IF=6.684, Q2), *Biomedicine & Pharmacotherapy* (IF=6.53, Q1), and *Cells* (IF=6.6, Q2) were emerging journals publishing papers in the field of autophagy and pancreatic cancer.

**Table 3:** The top 12 productive journals associated with autophagy of pancreatic cancer.

Rank	Journal	Count	Percentage	Total citations	IF (2020)	JCR division (2020)	Country
1	Cancers	23	3.7%	300	6.639	Q1	Switzerland
2	Oncotarget	19	3.0%	841	None	None	United States
3	Frontiers in Oncology	15	2.4%	211	6.244	Q2	Switzerland
4	Autophagy	13	2.1%	982	16.016	Q1	United States
5	International Journal of Molecular Sciences	13	2.1%	197	5.924	Q1	United States
6	Journal of Experimental & Clinical Cancer Research	12	1.9%	365	11.161	Q1	Italy
7	Oncology Reports	11	1.8%	282	3.906	Q3	Greece
8	Cell Death & Disease	10	1.6%	430	8.469	Q1	England
9	Scientific Reports	10	1.6%	370	4.38	Q1	England
10	Gastroenterology	9	1.5%	713	22.682	Q1	United States
10	Cancer Letters	9	1.5%	223	8.679	Q1	Netherlands
10	Plos One	9	1.5%	271	3.24	Q2	United States

Among 2719 co-cited journals, 14 journals had citations more than 500. As is shown in **Table 4**, *Nature* had the most co-citations up to 1415 times, followed by *Cancer Research* (1267 times), *Autophagy* (1238 times), and *Cell* (1116 times). Among the top 10 co-cited journals, 80% (8/10) had an IF of more than ten, 90% (9/10) were at the Q1 JCR division, and 80% (8/10) were from the United States.

**Table 4:** The most co-cited journals associated with autophagy of pancreatic cancer.

Rank	Journal	Total citations	IF (2020)	JCR division (2020)	Country
1	Nature	1415	49.962	Q1	England
2	Cancer Research	1267	12.701	Q1	United States
3	Autophagy	1238	16.016	Q1	United States
4	Cell	1116	41.584	Q1	United States
5	Journal of Biological Chemistry	865	5.157	Q2	United States
6	Proceedings of the National Academy of Sciences of the United States of America	825	11.205	Q1	United States
7	Clinical Cancer Research	723	12.531	Q1	United States
8	Genes & Development	708	11.361	Q1	United States
9	Oncogene	695	9.867	Q1	England
10	Cancer Cell	665	31.743	Q1	United States

### Analysis of authors and co-cited authors

**Figure 5:**
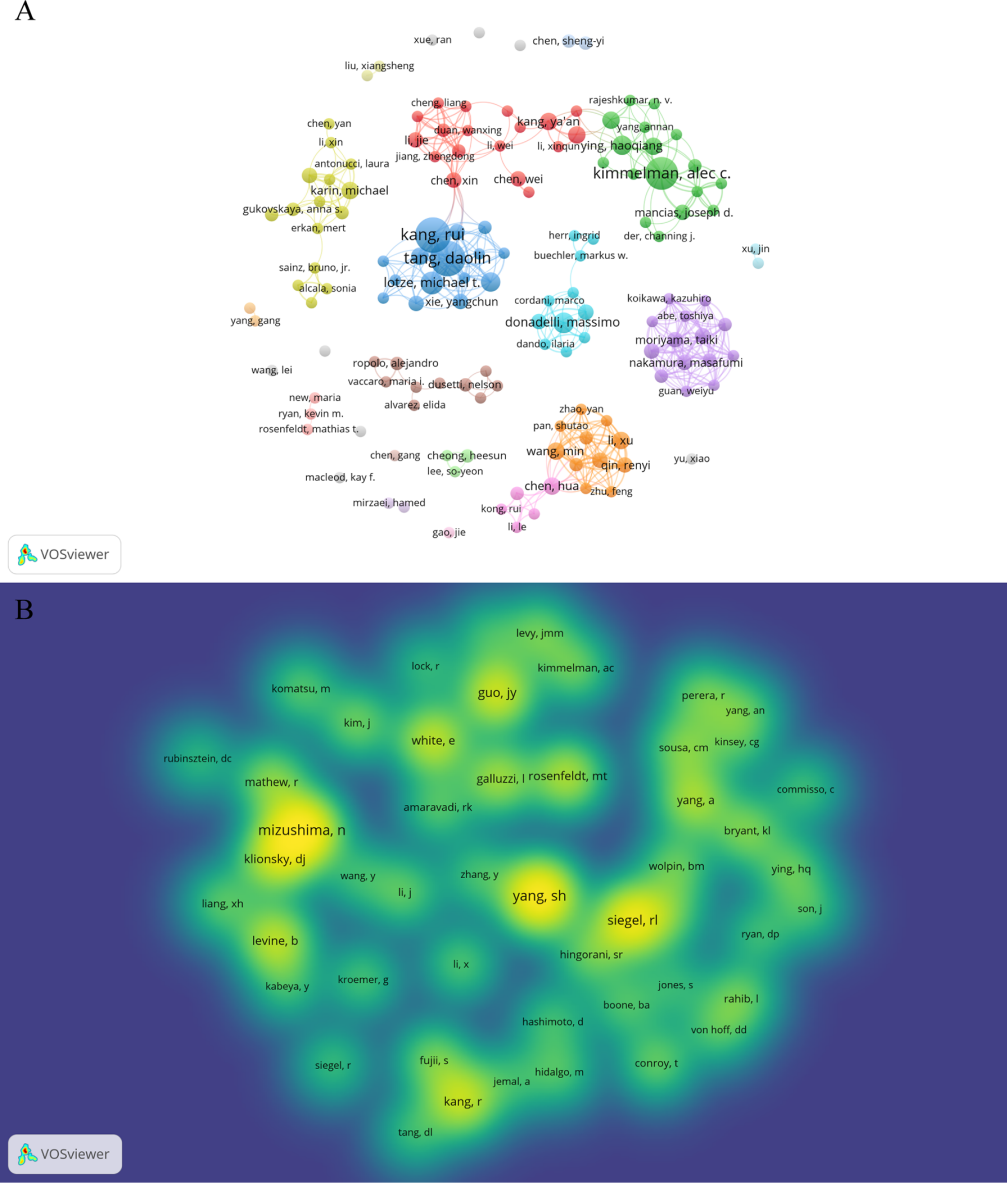
Authors and co-cited authors in the field of autophagy and pancreatic cancer. **(A)** The co-authorship network analysis of authors. The layout parameters: Attraction: 3, Repulsion: -1. The circle size means the number of publications; the thickness of the line means the strength of the connection; the circle colors mean different clusters; **(B)** The density map of co-cited authors. The layout parameters: Attraction: 2, Repulsion: 1. The color brightness means the frequency of occurrence.

A total of 3,993 authors contributed the 616 included publications. The top author is defined as one who has published at least 5 papers and received over 600 citations. Finally, ten top authors were identified (**Table 5**). By the number of papers, Daolin Tang and Rui Kang published the most papers (n=18, respectively), followed by Alec C Kimmelman (n=16), Michael T Lotze (n=9), and Haoqiang Ying (n=7). Papers published by Alec C Kimmelman who comes from New York University had the highest total number of citations (3288 times). Notably, top authors all come from the United States. The authors (n=142) who had published with a minimum of three publications were entered into co-authorship network analysis of authors (**Figure 5A**). There were strong collaborations among authors who were in the same cluster/color, such as Daolin Tang, Rui Kang, and Michael T Lotze. However, sparse connection was observed among different clusters, indicating little cooperation between research groups.

**Table 5:** The top 10 authors and most co-cited authors in the field of autophagy and pancreatic cancer.

Rank	Author	Count	Total citations	Institution	Location	Rank	Co-cited author	Total citations
1	Daolin Tang	18	1326	UT Southwestern Medical Center	United States	1	SH Yang	227
1	Rui Kang	18	1326	UT Southwestern Medical Center	United States	2	N Mizushima	222
3	Alec C Kimmelman	16	3288	New York University	United States	3	RL Siegel	176
4	Michael T Lotze	9	923	University of Pittsburgh	United States	4	JY Guo	140
5	Haoqiang Ying	7	1799	University of Texas MD Anderson Cancer Center	United States	5	R Kang	128
6	Herbert J Zeh	6	778	University of Pittsburgh	United States	6	E White	125
7	Xiaoxu Wang	6	2244	Dana-Farber Cancer Institute	United States	7	DJ Klionsky	125
8	Anirban Maitra	6	687	Johns Hopkins University	United States	8	B Levine	122
9	Yangchun Xie	5	796	University of Pittsburgh	United States	9	MT Rosenfeldt	112
10	Joseph D Mancias	5	827	Dana-Farber Cancer Institute	United States	10	A Yang	104

A total of 20,319 authors were co-cited at least once. There were 50 authors who had been co-cited with a minimum of 40 times. They were included to make the density visualization which can intuitively display the most co-cited authors (**Figure 5B**). Specifically, SH Yang (n=227) was the most frequent co-cited authors, followed by N Mizushima (n=222) and RL Siegel (n=176). The remaining seven top authors were co-cited from 104 to 140 (**Table 5**).

### Analysis of papers and co-cited references

Among the 616 papers in our study, 100 papers were cited more than 50 times. The most cited papers were summarized in **Table 6**. Four original articles by Shenghong Yang et al. (28), Andrea Viale et al. (39), Wen Hou et al. (40), and Cristovão M Sousa (41), with 957, 699, 594, and 531 citations, respectively, were ranked first, second, third and fourth. The remaining six studies [Mathias T Rosenfeldt 2013 (42); Rushika M Perera 2015 (43); Kirsten L Bryant 2014 (44), Jennifer A Kashatus 2015 (45), Annan Yang 2014 (46), and Conan G Kinsey 2019 (47)] were cited between 266 to 485 times. Also, four top cited articles were published in *Nature*.

**Table 6:** The most cited papers in the field of autophagy and pancreatic cancer.

Rank	Title	First author	Source	Type	Publication year	Total citations
1	pancreatic cancers require autophagy for tumor growth	Shenghong Yang	Genes & Development	Article	2011	957
2	Oncogene ablation-resistant pancreatic cancer cells depend on mitochondrial function	Andrea Viale	Nature	Article	2014	699
3	Autophagy promotes ferroptosis by degradation of ferritin	Wen Hou	Autophagy	Article	2016	594
4	Pancreatic stellate cells support tumour metabolism through autophagic alanine secretion	Cristovão M Sousa	Nature	Article	2016	531
5	p53 status determines the role of autophagy in pancreatic tumour development	Mathias T Rosenfeldt	Nature	Article	2013	485
6	Transcriptional control of autophagy-lysosome function drives pancreatic cancer metabolism	Rushika M Perera	Nature	Article	2015	453
7	KRAS: feeding pancreatic cancer proliferation	Kirsten L Bryant	Trends in Biochemical Sciences	Review	2014	413
8	Erk2 phosphorylation of Drp1 promotes mitochondrial fission and MAPK-driven tumor growth	Jennifer A Kashatus	Molecular Cell	Article	2015	347
9	Autophagy is critical for pancreatic tumor growth and progression in tumors with p53 alterations	Annan Yang	Cancer Discovery	Article	2014	305
10	Protective autophagy elicited by RAF→MEK→ERK inhibition suggests a treatment strategy for RAS-driven cancers	Conan G Kinsey	Nature Medicine	Article	2019	266

The results showed a total of 28,152 references were co-cited from 1 to 197. As is shown in **Table 7**, the most co-cited paper in the field of autophagy and pancreatic cancer by Shenghong Yang et al. (28), Mathias T Rosenfeldt et al. (42), and Annan Yang et al. (46), with 197, 96, and 86 citations, respectively, were ranked first, second, and third. The remaining eight top papers were co-cited from 54 to 73. Notably, the top 1 cited and co-cited paper was the same article published in *Genes & Development* by Shenghong Yang et al. in 2011 (28), entitled “Pancreatic cancers require autophagy for tumor growth”, indicating a wide influence and a highly proven peer recognition in the field.

**Table 7:** The most co-cited papers in the field of autophagy and pancreatic cancer.

Rank	Title	First author	Source	Type	Publication year	Total citations
1	Pancreatic cancers require autophagy for tumor growth	Shenghong Yang	Genes & Development	Article	2011	197
2	p53 status determines the role of autophagy in pancreatic tumour development	Mathias T Rosenfeldt	Nature	Article	2013	96
3	Autophagy is critical for pancreatic tumor growth and progression in tumors with p53 alterations	Annan Yang	Cancer Discovery	Article	2014	86
4	Activated Ras requires autophagy to maintain oxidative metabolism and tumorigenesis	Jessie Yanxiang Guo	Genes & Development	Article	2011	73
5	Autophagy is activated in pancreatic cancer cells and correlates with poor patient outcome	Satoshi Fujii	Cancer Science	Article	2008	70
6	Pancreatic stellate cells support tumour metabolism through autophagic alanine secretion	Cristovão M Sousa	Nature	Article	2016	66
7	Transcriptional control of autophagy-lysosome function drives pancreatic cancer metabolism	Rushika M Perera	Nature	Article	2015	66
8	Projecting cancer incidence and deaths to 2030: the unexpected burden of thyroid, liver, and pancreas cancers in the United States	Lola Rahib	Cancer Research	Article	2014	61
9	Cancer statistics, 2019	Rebecca L Siegel	CA-A Cancer Journal for Clinicians	Article	2019	56
10	Autophagy Sustains Pancreatic Cancer Growth through Both Cell-Autonomous and Nonautonomous Mechanisms	Annan Yang	Cancer Discovery	Article	2018	54
10	Phase II and pharmacodynamic study of autophagy inhibition using hydroxychloroquine in patients with metastatic pancreatic adenocarcinoma	Brian M Wolpin	Oncologist	Article	2014	54

### Analysis of keyword co-occurrence

**Figure 6:**
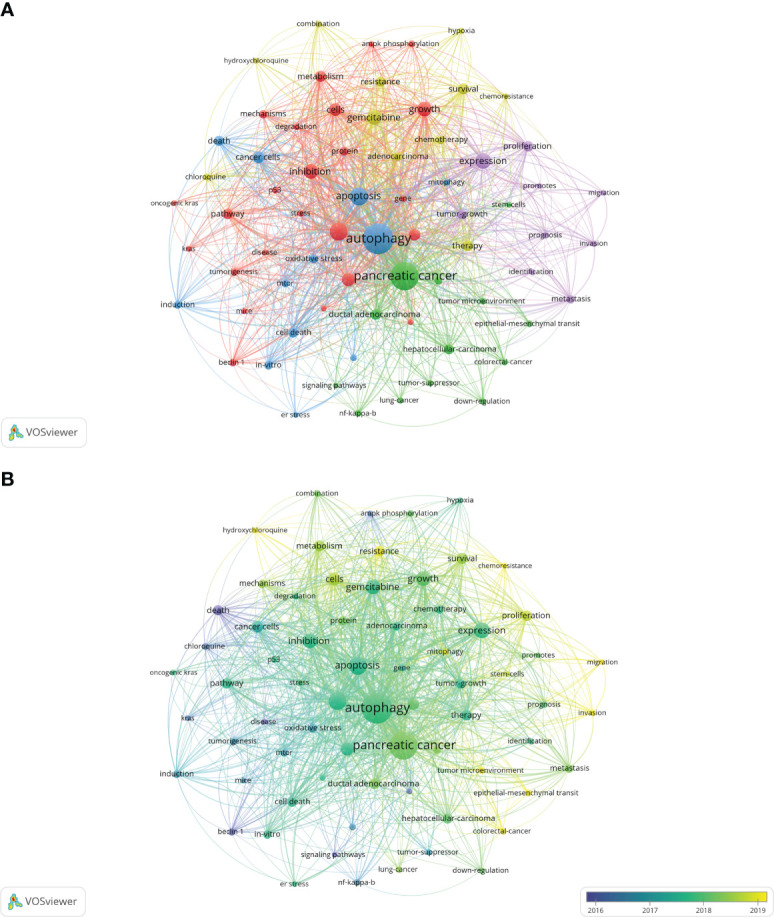
Keywords related to autophagy of pancreatic cancer. **(A)** Network visualization of keywords drawn by VOSviewer. The layout parameters: Attraction: 2, Repulsion: 1. The circle size means the frequency of occurrence; the circle colors mean different clusters; **(B)** Overlay visualization of keywords drawn by VOSviewer. The layout parameters: Attraction: 2, Repulsion: 1. The circle size means the frequency of occurrence; the circle colors mean the average published year.

The co-occurrence analysis of all keywords showed a total of 2668 keywords were extracted. The keywords with the same meaning were merged (**Supplemental File S2**), such as pancreatic cancer, cancer cells, beclin 1, etc. **Table 8** listed the top 20 high-frequency co-occurrence keywords. These keywords may reveal the hotspots in the field of autophagy and pancreatic cancer. The most co-occurrence keyword was autophagy (n=419 co-occurrences), followed by pancreatic cancer (n=360), apoptosis (n=146), cancer (n=146), expression (n=111), growth (n=102), gemcitabine (n=101), inhibition (n=97), activation (n=80), cells (n=75), etc. According to Price’s Law, the keywords appeared over 15 times were used to build a network map to visualize keyword clusters (**Figure 6A**) and 69 keywords were finally identified. The network was divided into five clusters with different colors and it is highly homogeneous between the items in the same color. There were 24 items in cluster 1 (red), including activation, cancer, cells, growth, inhibition, metabolism, pathway, progression, degradation, inflammation, mechanisms, tumorigenesis, AMPK, beclin 1, mice, KRAS, p53, p62, phosphorylation, stress, etc. There were 13 items in cluster 2 (green), including tumor microenvironment, epithelial-mesenchymal transition, down-regulation, NF-kappa-B, stem-cells, tumor-suppressor, etc. There were 12 items in cluster 3 (blue), including apoptosis, cell death, death, endoplasmic-reticulum stress, *in-vitro*, induction, mitophagy, mTOR, oxidative stress, etc. There were 11 items in cluster 4 (yellow), including gemcitabine, survival, resistance, chemoresistance, chemotherapy, chloroquine, combination, hydroxychloroquine, hypoxia, therapy, etc. There were 9 items in cluster 5 (purple), including expression, proliferation, tumor-growth, identification, prognosis, invasion, metastasis, migration, and promotes.

**Table 8:** The top 20 keywords related to autophagy of pancreatic cancer.

Rank	keyword	Count	Rank	keyword	Count
1	autophagy	419	11	cancer cells	64
2	pancreatic cancer	360	12	progression	61
3	apoptosis	146	13	survival	55
3	cancer	146	13	metabolism	55
5	expression	111	15	proliferation	51
6	growth	102	15	therapy	51
7	gemcitabine	101	15	pathway	51
8	inhibition	97	15	ductal adenocarcinoma	51
9	activation	80	19	resistance	49
10	cells	75	20	death	48

The overlay visualization map of the 69 keywords is showed in **Figure 6B**. The research focus can be intuitively observed by the evolution of high-frequency keywords over time. The yellow nodes represented the emerging keywords near 2019. Among which, the most co-occurrence keywords were resistance (n=49 co-occurrences), followed by tumor microenvironment (n=23), epithelial-mesenchymal transition (EMT) (n=22), mitophagy (n=21), and invasion (n=21). These keywords may become the future research hotspots in the field of autophagy and pancreatic cancer.

## Discussion

In this study, we used VOSviewer software to perform a bibliometric analysis based on the literature related to autophagy of pancreatic cancer in WoSCC database from 2011 to 2022 (June 9th, 2022). A total of 616 studies were written by 3993 authors, covered 45 countries and 871 organizations, published in 263 journals and co-cited 28152 references from 2719 journals. Most of which are original articles (77.8%). An average of 45.70 references each publication were noted. The primary aim of the current study was to explore the global research features and hotspots and forecast the emerging trends which may be helpful to researchers in autophagy of pancreatic cancer field.

Overall, the annual publication output has dramatically increased from 11 in 2011 up to 101 in 2021 which reveals that attention has been increasing in autophagy of pancreatic cancer field over the past 12 years. Autophagy plays an important role in tumor pathogenesis and contributes to tumor growth (48, 49). The article published in *Genes & Development* (IF=11.361) by Shenghong Yang et al, in 2011 which confirmed that pancreatic cancers actually require autophagy for tumorgenic growth has been cited and co-cited the most frequently (28), indicating Shenghong Yang is an accomplished scholar in this field and his study is considered as the most fundamental and important study. Besides, it pointed chloroquine and its derivatives are powerful inhibitors of autophagy which could be used to treat pancreatic cancer patients (28). Therefore, more attention on the research of autophagy and pancreatic cancer field will be triggered (50).

As far as countries for publication of papers are considered, a bibliometric analysis of autophagy showed that China and the United States were the most productive countries (27). Again, one bibliometric study on mitophagy (34) and the other bibliometric study on pancreatic cancer research (35) arrived the same conclusion. Our results also showed that China and the United States were the most frequent publishers in the field of autophagy and pancreatic cancer. 76.5% of the total publications was contributed by China and the United States, far more than any other country. This phenomenon could be called “Matthew effect”. In the network visualization map, extensive cooperation was observed between countries with a minimum of five publications, indicating a widespread trans-national communication in the research of autophagy and pancreatic cancer. Specifically, China and the United States collaborated closely. The United States can play as the central role in the cooperation network map with its cooperation with 23 countries. Despite the United States, Germany, Spain, China, Italy, and England can also be suggested as minor cores as they cooperated with 12, 11, 10, 10, and 10 countries, respectively. However, China had a lower average number of citations than United States, Italy, Germany, England, France and Japan, and none of the top 10 cited and top 11 co-cited papers were written by Chinese scholars, implying the quality of studies written by Chinese scholars needs further improvement. In terms of organizations, China and the United States contributed six and five of the top 11 organizations, respectively. The number of average citations per papers of top 11 organizations was generally consistent with that of countries. Among which, Dana-Farber Cancer Institute had the highest average number of citations (169.58 times) among the top 11 organizations, and two high-frequency cited authors (Xiaoxu Wang and Joseph D Mancias) were from this institution, showing the high influence of its published articles. Besides, cooperation between countries were found to be a little sparser than those between agencies, indicating that international cooperation should be strengthened in this field. Notably, University of Texas MD Anderson Cancer Center, the most productive organization, collaborated most closely with many United States universities and research institutions, and also with Universities from China, such as China Medical University, Fudan University, Sun Yat-Sen University, Xi’an Jiaotong University, and Tongji University, showing that the United States and China collaborated closely between organizations.

When it comes to journals and co-cited journals, our results showed the journals published the most papers related to autophagy of pancreatic cancer were *Cancers* (n=23), *Oncotarget* (n=19), *Frontiers in Oncology* (n=15), *Autophagy* (n=13), and *International Journal of Molecular Sciences* (n=13). Among the top 12 journals, 66.7% had an IF of more than five, and 66.7% were at the Q1 JCR division. *Nature* (n=1415 times), *Cancer Research* (n=1267 times), *Autophagy* (n=1238 times), and *Cell* (n=1116 times) were the most high-frequency co-cited journals. Among the top 10 co-cited journals, 80% had an IF of more than ten, 90% were at the Q1 JCR division. These data indicated many high-quality and high-impact journals were particularly interested in and play a significant role in the field of autophagy and pancreatic cancer. Besides, it is worth noting that *Cancers*, the most productive journal, was also an emerging journal in recent 3 years, implying this journal was very pleasure to accept the researches in this field. Despite the most productive journals, *Frontiers in Cell and Developmental Biology* (IF=6.684, Q2), *Biomedicine & Pharmacotherapy* (IF=6.53, Q1), and *Cells* (IF=6.6, Q2) were the emerging journals that accepted related papers in recent 3 years. These results will also assist future scholars in selecting journals when submitting manuscripts associated to autophagy of pancreatic cancer.

A high citation frequency indicating a wide influence and a highly proven peer recognition in the field. In this bibliometric analysis, the top 10 most-cited papers were as follows (**Table 6**): Shenghong Yang et al. published “pancreatic cancers require autophagy for tumor growth (28)” in *Genes & Development* in 2011, which was the most cited paper (957 citations). This study reported that pancreatic cancers have a distinct dependence on autophagy. The second cited paper, “Oncogene ablation-resistant pancreatic cancer cells depend on mitochondrial function”, was published by Andrea Viale et al. (39) in *Nature* in 2014. This study illuminated a therapeutic strategy of combined targeting of the KRAS pathway and mitochondrial respiration to treat pancreatic cancer. The third cited paper, “Autophagy promotes ferroptosis by degradation of ferritin” was published by Wen Hou et al. (40) in *Autophagy* in 2016. This study found autophagy promotes ferroptosis by degradation of ferritin which provide novel insight into the interplay between autophagy and regulated cell death. The fourth cited paper was published by Cristovão M Sousa et al. (41) in *Nature* in 2016. It reported pancreatic stellate cells (PSCs)-derived alanine is an alternative fuel source that can sustain the growth of cancer cells in the tumor microenvironment. And alanine release in PSC is dependent on PSC autophagy which is mediated by cancer cells. The fifth cited paper was published by Mathias T Rosenfeldt et al. (42) in *Nature* in 2013. It showed the progression of pancreatic cancer is intrinsically associated with the status of p53 (a tumor suppressor gene). Inhibition of autophagy promotes cancer onset instead of blocking cancer progression in mouse model with oncogenic KRAS but without p53. The sixth cited paper was published by Rushika M Perera et al. (43) in *Nature* in 2015. This article reported MiT/TFE-dependent autophagy-lysosome activation is essential for pancreatic cancer growth, which is a novel hallmark of malignant tumor. The seventh cited paper was published by Turtle et al. (44) Kirsten L Bryant in *Trends in Biochemical Sciences* in 2014. This is a review presented oncogenic KRAS plays a critical role in controlling tumor metabolism by increasing autophagy and orchestrating other multiple metabolic changes. The eighth cited paper was published by Jennifer A Kashatus et al. (45) in *Molecular Cell* in 2015. This article illuminated the activation of Ras or MAPK pathway (the downstream biological process) leads to Mek-dependent phosphorylation of the GTPase Drp1 and subsequent mitochondrial fission. Inhibition of Drp1 or its phosphorylation blocks pancreatic cancer growth. The ninth cited paper was published by Annan Yang et al. (46) in *Cancer Discovery* in 2014. This article reported autophagy plays a central role in pancreatic cancer and showed that autophagy inhibition may have therapeutical effect on pancreatic cancer, independent of p53 status. The tenth cited paper was published by Conan G Kinsey et al. (47) in *Nature Medicine* in 2019. This article represented trametinib combined with hydroxychloroquine may be a new strategy to treat RAS-driven cancers. Besides, the most co-cited papers were listed in **Table 7**. These most co-cited studies have a major impact on autophagy of pancreatic cancer field. The first, second, third, sixth and seventh co-cited papers are the same as the first, fifth, ninth, fourth and sixth cited paper listed in **Table 6**. The eighth and ninth co-cited papers are about the epidemiology of cancers. The remaining 4 top co-cited articles are mainly about the role of autophagy in pancreatic cancer. Keywords represent the major topic of papers. To explore the global research features and hotspots, we constructed a co-occurrence analysis of all keywords in the field of autophagy and pancreatic cancer by VOSviewer. As autophagy broadly consists of macroautophagy, microautophagy, and chaperone-mediated autophagy, we individually searched for publications concerning the three types. The publication numbers were 18, 0, and 7, respectively. The content of the remaining publications was indistinguishable. Macroautophagy has been studied the most. The keywords appeared over 15 times were clustered into five main categories in the network visualization map (**Figure 6A**) which can intuitively show the direction and scope in this field. After reviewing and summarizing relevant researches, we found the keywords in cluster 1 (red) and cluster 5 (purple) mainly focused on the regulation mechanisms of autophagy in pancreatic cancer onset and progression. Among which, expression, growth, and inhibition could represent the research hotspots. In the top cite and co-cited papers, Shenghong Yang et al. reported pancreatic cancers required autophagy for tumor growth in 2011 (28), which is considered as the most fundamental and important study in this field. The other article published in *Genes & Development* by Jessie Yanxiang Guo et al. in 2011, reported activated oncogene HRAS or KRAS could increase basal autophagy which was essential to maintain human cancer cell survival in starvation and in oncogenesis (51). As KRAS mutation was found in 70~95% of PDAC patients (52), researches on the regulation of autophagy in Ras-expressing pancreatic cancer cells were rapidly increasing. Notably, Mathias T Rosenfeldt et al. showed Inhibition of autophagy promotes cancer onset instead of blocking cancer progression in mouse model with oncogenic KRAS but without p53 (42), suggesting a dual role of autophagy in pancreatic cancer progression (53, 54). In the transcriptional program, Rushika M Perera et al. presented MiT/TFE-dependent autophagy-lysosome activation is essential for pancreatic cancer growth, which is a novel hallmark of malignant tumor (43). Besides, Di Malta, C. et al. found transcriptional activation of Rag guanosine triphosphatases could control the mechanistic target of rapamycin complex 1 and regulate anabolic pathways related to nutrient metabolism, leading to excessive cell proliferation and tumor growth (55). Researches have also shown that autophagy supports the growth of pancreatic cancer through both cell-autonomous and nonautonomous pathways (56). These studies provide us insights into the role of autophagy in pancreatic cancer, which may be used to treat this malignant cancer in future. The keywords in cluster 2 (green) were mainly associated with the relationship between autophagy and tumor microenvironment as well as that between autophagy and EMT in pancreatic cancer. Notably, tumor microenvironment and EMT were the emerging keywords in recent years, indicating they may become the future hotspots in the field of autophagy and pancreatic cancer. Hypoxic tumor microenvironment is characterized as a hallmark of pancreatic cancer (57). Increased autophagy flux may mediate the survival of pancreatic cancer stem cells (CSCs) under a hypoxic tumor microenvironment. The inhibition of autophagy converts survival signaling to suicide and finally suppresses cancer development in mouse models (58). Besides, a top cited and co-cited paper published in Nature by Cristovão M Sousa et al. in 2016, reported PSCs-derived alanine is an alternative fuel source that can sustain the growth of cancer cells in the tumor microenvironment. And alanine release in PSC is dependent on PSC autophagy which is mediated by cancer cells (41). Despite of CSC and PSC, immune cells, endothelial cells, and fibroblasts may also promote tumor progression through the metabolic crosstalk with malignant cells in the tumor microenvironment (59), the role of autophagy in tumor microenvironment needs further study. In terms of EMT, it is a trans-differentiation process in which epithelial cells acquire mesenchymal features that promote the invasion and metastasis of cancers (60). Enhanced autophagy induced by HIF-1 alpha was reported to promote EMT and the metastatic ability of pancreatic CSCs (61). In RAS-mutated pancreatic cancer cells, the inhibition of autophagy activated the SQSTM1/p62-mediated NF-kappa-B pathway, subsequently enhancing EMT which finally promoted cancer invasion (62). This broadens the horizon for the research of the dual role of autophagy in pancreatic cancer. The keywords in cluster 3 (blue) were mainly related to the role of autophagy in the apoptosis of pancreatic cancer cells. Apoptosis represents a type of programmed cell death that can remove the damaged cells orderly and efficiently (63). Targeting apoptosis is a common therapy strategy for PDAC. However, the cancer cells can establish various mechanisms to reduce apoptosis, including autophagy (64). For example, mitochondrial uncoupling protein 2 (UCP2) plays an essential role in tumorigenesis and development. UCP2 induces autophagy through enhancing Beclin 1 and inhibiting the AKT-MTOR pathway, leading to anti-apoptosis effects or inhibiting other types of cell death in a reactive oxygen species (ROS)-dependent mechanism (65), implying an anti-apoptosis role of autophagy. Eicosapentaenoic acid, a common omega-3 fatty acid, can not only induce autophagy but impair its anti-apoptosis ability in pancreatic cancer cells (66). Ubiquitin specific peptidase 22 (USP22) is an epigenetic regulator, it was reported USP22 induced autophagy by activating MAPK1, thereby promoting cell proliferation and gemcitabine resistance in pancreatic cancer cell lines (67). As discussed above, CSCs and PSCs sustain tumor growth depend on autophagy. Studies have reported inhibiting autophagy also triggers apoptosis in CSCs and PSCs (58, 68). These studies indicate that chemotherapy combined the regulation of autophagy could be a potential future direction in treating pancreatic cancers. Besides, mitophagy is an emerging keyword in this cluster. It is reported mitophagy involved the cell death and modulation of metabolism in pancreatic cancer. Again, mitophagy plays a double-edged action in the regulation of the antitumor efficacy of certain cytotoxic agents (69). The keywords in cluster 4 (yellow) were mainly about the autophagy regulation in the treatment of pancreatic cancer. Autophagy is an essential catabolic mechanism in pancreatic cancer onset and progression. The inhibitions of autolysosome formation, a lysosomotropic agent named chloroquine (CQ) and a V-ATPase inhibitor named bafilomycin A_1_, were reported to suppress tumorigenic growth of pancreatic cancers alone (28). However, a phase II and pharmacodynamic study showed hydroxychloroquine (HCQ, an inhibitor of autophagy) monotherapy did not result in a consistent autophagy inhibition as evaluated by peripheral lymphocytes LC3-II levels and achieved negligible benefits in previously treated patients with metastatic pancreatic cancer (70). The dual role of autophagy in pancreatic cancer makes it difficult to be a therapeutic target alone (54). Therefore, most studies focused on combination therapy for treating pancreatic cancer by inhibiting [e.g., CQ or bafilomycin A_1_ (28), DQ661 (71)] or inducing [Quercetin (72), Demethylzeylasteral (73)] autophagy to increase therapeutic efficacy of gemcitabine or other antitumor drugs. As mentioned above, activation of autophagy has led to gemcitabine resistance by inhibiting apoptosis in the treatment of PDAC patients. A recent randomized phase II preoperative study reported resectable pancreatic adenocarcinoma patients treated by gemcitabine and nab-paclitaxel with HCQ resulted in an evidence of autophagy inhibition and immune activity and achieved greater pathologic tumor response and lower CA199 levels than patients treated by gemcitabine and nab-paclitaxel alone (74). Alternatively, autophagy induction may result in an antitumor efficacy through autophagy-mediated metabolic stress or injury. For instance, combined therapy with Demethylzeylasteral and gemcitabine induces autophagic cell death and demethylzeylasteral could increases the chemosensitivity to gemcitabine in treating pancreatic cancer (73). These results suggest autophagy-related drugs play a complex role in pancreatic cancer chemotherapy. As discussed above, the current research related to autophagy of pancreatic cancer mainly about basic research and clinical application. The focus of scholars has gradually switched from basic research to clinical application. The hot topics in current research have always been the mechanisms of autophagy in tumor onset and progression, the role of autophagy in tumor apoptosis, and autophagy-related drugs in treating pancreatic cancer (especially combined therapy). The emerging topics mainly focused on chemotherapy resistance mediated by autophagy, tumor microenvironment related to autophagy, autophagy-depended EMT, mitophagy, and the role of autophagy in tumor invasion, that may become the main future direction in the field of autophagy and pancreatic cancer.

Our study first conducted a bibliometric analysis related to autophagy of pancreatic cancer, providing an objective and intuitive evaluation of the research features and hotspots and forecasting the emerging trends in that field. Admittedly, this study has some limitations. First, we collected the literature data only from WOSCC database and the related papers from other sources may be neglected. Secondly, the literature language was limited to English, that may result in the source of bias. Thirdly, since the total number of citations depends on various factors (e.g., time of publication, journal, research area), the number of citations may not accurately represent the impact of a paper, and some recent landmark papers may have been omitted.

## Conclusion

This study showed research activities were multiplying in the field of autophagy and pancreatic cancer. China and the United States were the most frequent publishers and collaborated closely in this field. *Cancers* published most papers in this field and was very pleasure to accept the related researches. We have also listed the most cited and co-cited papers and authors. Importantly, the mechanisms of autophagy in tumor onset and progression, the role of autophagy in tumor apoptosis, and autophagy-related drugs in treating pancreatic cancer (especially combined therapy) were the research hotspots. The emerging topics were chemotherapy resistance mediated by autophagy, tumor microenvironment related to autophagy, autophagy-depended EMT, mitophagy, and the role of autophagy in tumor invasion. These results undoubtedly provide scholars with new clues and ideas in the field of autophagy and pancreatic cancer.

## Data availability statement

The original contributions presented in the study are included in the article/**Supplementary Material**. Further inquiries can be directed to the corresponding authors.

## Author contributions

XLO and CG designed the project. MYS, QL and XLO collected the literature data. MYS, QL and MX contributed to the main part of data analysis and prepared the main manuscript. MX, YJL and YWZ revised the article and made suggestions to improve the article. YJL and YWZ prepared the figures and tables. Final approval of manuscript: All authors.

## Funding

This work was supported by the National Natural Science Foundation of China (No. 81802378) and the Gusu Health Talents Training Project (No. GSWS2021058).

## Conflict of interest

The authors declare that the research was conducted in the absence of any commercial or financial relationships that could be construed as a potential conflict of interest.

## Publisher’s note

All claims expressed in this article are solely those of the authors and do not necessarily represent those of their affiliated organizations, or those of the publisher, the editors and the reviewers. Any product that may be evaluated in this article, or claim that may be made by its manufacturer, is not guaranteed or endorsed by the publisher.

## References

[1] Siegel RL Miller KD Fuchs HE Jemal A. Cancer statistics, 2022. CA Cancer J Clin (2022) 72(1):7–33. doi: 10.3322/caac.21708 35020204

[2] Neoptolemos JP Kleeff J Michl P Costello E Greenhalf W. Therapeutic developments in pancreatic cancer: current and future perspectives. Nat Rev Gastroenterol Hepatol (2018) 15(6):333–48. doi: 10.1038/s41575-018-0005-x 29717230

[3] Mizrahi JD Surana R Valle JW Shroff RT. Pancreatic cancer. Lancet (2020) 395(10242):2008–20. doi: 10.1016/S0140-6736(20)30974-0 32593337

[4] Cuervo AM. Autophagy: in sickness and in health. Trends Cell Biol (2004) 14(2):70–7. doi: 10.1016/j.tcb.2003.12.002 15102438

[5] Goswami SK Das DK. Autophagy in the myocardium: Dying for survival? Exp Clin Cardiol (2006) 11(3):183–8. PMC2276148 18651029

[6] Klionsky DJ. Autophagy: from phenomenology to molecular understanding in less than a decade. Nat Rev Mol Cell Biol (2007) 8(11):931–7. doi: 10.1038/nrm2245 17712358

[7] Levine B. Cell biology: autophagy and cancer. Nature (2007) 446(7137):745–7. doi: 10.1038/446745a 17429391

[8] Levine B Klionsky DJ. Development by self-digestion: molecular mechanisms and biological functions of autophagy. Dev Cell (2004) 6(4):463–77. doi: 10.1016/s1534-5807(04)00099-1 15068787

[9] Levine B Kroemer G. Autophagy in the pathogenesis of disease. Cell (2008) 132(1):27–42. doi: 10.1016/j.cell.2007.12.018 18191218 PMC2696814

[10] Mizushima N Yoshimori T Ohsumi Y. The role of atg proteins in autophagosome formation. Annu Rev Cell Dev Biol (2011) 27:107–32. doi: 10.1146/annurev-cellbio-092910-154005 21801009

[11] Rogov V Dötsch V Johansen T Kirkin V. Interactions between autophagy receptors and ubiquitin-like proteins form the molecular basis for selective autophagy. Mol Cell (2014) 53(2):167–78. doi: 10.1016/j.molcel.2013.12.014 24462201

[12] Shintani T Klionsky DJ. Autophagy in health and disease: a double-edged sword. Science (2004) 306(5698):990–5. doi: 10.1126/science.1099993 PMC1705980 15528435

[13] Mizushima N. A brief history of autophagy from cell biology to physiology and disease. Nat Cell Biol (2018) 20(5):521–7. doi: 10.1038/s41556-018-0092-5 29686264

[14] Marinković M Šprung M Buljubašić M Novak I. Autophagy modulation in cancer: Current knowledge on action and therapy. Oxid Med Cell Longev (2018) 2018:8023821. doi: 10.1155/2018/8023821 29643976 PMC5831833

[15] Nakatogawa H. Mechanisms governing autophagosome biogenesis. Nat Rev Mol Cell Biol (2020) 21(8):439–58. doi: 10.1038/s41580-020-0241-0 32372019

[16] Jin M Liu X Klionsky DJ. SnapShot: Selective autophagy. Cell (2013) 152(1-2):368–8. doi: 10.1016/j.cell.2013.01.004 PMC3627723 23332767

[17] Gatica D Lahiri V Klionsky DJ. Cargo recognition and degradation by selective autophagy. Nat Cell Biol (2018) 20(3):233–42. doi: 10.1038/s41556-018-0037-z PMC6028034 29476151

[18] Johansen T Lamark T. Selective autophagy: ATG8 family proteins, LIR motifs and cargo receptors. J Mol Biol (2020) 432(1):80–103. doi: 10.1016/j.jmb.2019.07.016 31310766

[19] Kirkin V. History of the selective autophagy research: How did it begin and where does it stand today? J Mol Biol (2020) 432(1):3–27. doi: 10.1016/j.jmb.2019.05.010 31082435 PMC6971693

[20] Schuck S. Microautophagy - distinct molecular mechanisms handle cargoes of many sizes. J Cell Sci (2020) 133(17):jcs246322. doi: 10.1242/jcs.246322 32907930

[21] Kaushik S Cuervo AM. The coming of age of chaperone-mediated autophagy. Nat Rev Mol Cell Biol (2018) 19(6):365–81. doi: 10.1038/s41580-018-0001-6 PMC6399518 29626215

[22] Fujiwara Y Wada K Kabuta T. Lysosomal degradation of intracellular nucleic acids-multiple autophagic pathways. J Biochem (2017) 161(2):145–54. doi: 10.1093/jb/mvw085 28039390

[23] Dikic I Elazar Z. Mechanism and medical implications of mammalian autophagy. Nat Rev Mol Cell Biol (2018) 19(6):349–64. doi: 10.1038/s41580-018-0003-4 29618831

[24] Levine B Kroemer G. Biological functions of autophagy genes: A disease perspective. Cell (2019) 176(1-2):11–42. doi: 10.1016/j.cell.2018.09.048 30633901 PMC6347410

[25] Mizushima N Levine B. Autophagy in human diseases. N Engl J Med (2020) 383(16):1564–76. doi: 10.1056/NEJMra2022774 33053285

[26] Mizushima N Levine B Cuervo AM Klionsky DJ. Autophagy fights disease through cellular self-digestion. Nature (2008) 451(7182):1069–75. doi: 10.1038/nature06639 PMC2670399 18305538

[27] Hong T Feng X Tong W Xu W. Bibliometric analysis of research on the trends in autophagy. PeerJ (2019) 7:e7103. doi: 10.7717/peerj.7103 31205825 PMC6556104

[28] Yang SH Wang XX Contino G Liesa M Sahin E. Pancreatic cancers require autophagy for tumor growth. Genes Dev (2011) 25(7):717–29. doi: 10.1101/gad.2016111 PMC3070934 21406549

[29] Donthu N Kumar S Mukherjee D Pandey N Lim WM. How to conduct a bibliometric analysis: An overview and guidelines. J Business Res (2021) 133:285–96. doi: 10.1016/j.jbusres.2021.04.070

[30] Liu Y Xu Y Cheng X Lin Y Jiang S. Research trends and most influential clinical studies on anti-PD1/PDL1 immunotherapy for cancers: A bibliometric analysis. Front Immunol (2022) 13:862084. doi: 10.3389/fimmu.2022.862084 35493449 PMC9044908

[31] Zhang J Song L Xu L Fan Y Wang T. Knowledge domain and emerging trends in ferroptosis research: A bibliometric and knowledge-map analysis. Front Oncol (2021) 11:686726. doi: 10.3389/fonc.2021.686726 34150654 PMC8209495

[32] Shen J Shen H Ke L Chen J Dang X. Knowledge mapping of immunotherapy for hepatocellular carcinoma: A bibliometric study. Front Immunol (2022) 13:815575. doi: 10.3389/fimmu.2022.815575 35173728 PMC8841606

[33] Thompson DF Walker CK. A descriptive and historical review of bibliometrics with applications to medical sciences. Pharmacotherapy (2015) 35(6):551–9. doi: 10.1002/phar.1586 25940769

[34] Chen J Li X Jia Y Xia Z Ye J. Publication trends on mitophagy in the world and China: A 16-year bibliometric analysis. Front Cell Dev Biol (2021) 9:793772. doi: 10.3389/fcell.2021.793772 34912814 PMC8667272

[35] Wang K Herr I. Machine-Learning-Based bibliometric analysis of pancreatic cancer research over the past 25 years. Front Oncol (2022) 12:832385. doi: 10.3389/fonc.2022.832385 35419289 PMC8995465

[36] Wu K Liu Y Liu L Peng Y Pang H. Emerging trends and research foci in tumor microenvironment of pancreatic cancer: A bibliometric and visualized study. Front Oncol (2022) 12:810774. doi: 10.3389/fonc.2022.810774 35515122 PMC9063039

[37] Shi H Chen H Qian B Huang Z Tan P. The 100 most cited articles on pancreatic neuroendocrine tumors from 2000 to 2020: a bibliometric analysis. Jpn J Clin Oncol (2022) 52(3):251–9. doi: 10.1093/jjco/hyab205 34954797

[38] van Eck NJ Waltman L. Software survey: VOSviewer, a computer program for bibliometric mapping. Scientometrics (2010) 84(2):523–38. doi: 10.1007/s11192-009-0146-3 PMC2883932 20585380

[39] Viale A Pettazzoni P Lyssiotis CA Ying HQ Sanchez N. Oncogene ablation-resistant pancreatic cancer cells depend on mitochondrial function. Nature (2014) 514(7524):628–+. doi: 10.1038/nature13611 PMC4376130 25119024

[40] Hou W Xie YC Song XX Sun XF Lotze MT. Autophagy promotes ferroptosis by degradation of ferritin. Autophagy (2016) 12(8):1425–8. doi: 10.1080/15548627.2016.1187366 PMC4968231 27245739

[41] Sousa CM Biancur DE Wang XX Halbrook CJ Sherman MH. Pancreatic stellate cells support tumour metabolism through autophagic alanine secretion. Nature (2016) 536(7617):479–+. doi: 10.1038/nature19084 PMC5228623 27509858

[42] Rosenfeldt MT O'Prey J Morton JP Nixon C MacKay G. p53 status determines the role of autophagy in pancreatic tumour development. Nature (2013) 504(7479):296–+. doi: 10.1038/nature12865 24305049

[43] Perera R Stoykova S Nicolay BN Ross KN Fitamant J. Transcriptional control of autophagy-lysosome function drives pancreatic cancer metabolism. Nature (2015) 524(7565):361–U251. doi: 10.1038/nature14587 26168401 PMC5086585

[44] Bryant KL Mancias JD Kimmelman AC Der CJ. KRAS: feeding pancreatic cancer proliferation. Trends Biochem Sci (2014) 39(2):91–100. doi: 10.1016/j.tibs.2013.12.004 24388967 PMC3955735

[45] Kashatus JA Nascimento A Myers LJ Sher A Byrne FL. Erk2 phosphorylation of Drp1 promotes mitochondrial fission and MAPK-driven tumor growth. Mol Cell (2015) 57(3):537–51. doi: 10.1016/j.molcel.2015.01.002 PMC4393013 25658205

[46] Yang A Rajeshkumar NV Wang XX Yabuuchi S Alexander BM. Autophagy is critical for pancreatic tumor growth and progression in tumors with p53 alterations. Cancer Discov (2014) 4(8):905–13. doi: 10.1158/2159-8290.cd-14-0362 PMC4125497 24875860

[47] Kinsey CG Camolotto SA Boespflug AM Guillen KP Foth M. Protective autophagy elicited by RAF -> MEK -> ERK inhibition suggests a treatment strategy for RAS-driven cancers. Nat Med (2019) 25(4):620–+. doi: 10.1038/s41591-019-0367-9 PMC6452642 30833748

[48] Poillet-Perez L White E. Role of tumor and host autophagy in cancer metabolism. Genes Dev (2019) 33(11-12):610–9. doi: 10.1101/gad.325514.119 PMC6546058 31160394

[49] Ishaq M Ojha R Sharma AP Singh SK. Autophagy in cancer: Recent advances and future directions. Semin Cancer Biol (2020) 66:171–81. doi: 10.1016/j.semcancer.2020.03.010 32201367

[50] Li J Chen X Kang R Zeh H Klionsky DJ. Regulation and function of autophagy in pancreatic cancer. Autophagy (2021) 17(11):3275–96. doi: 10.1080/15548627.2020.1847462 PMC8632104 33161807

[51] Guo JY Chen HY Mathew R Fan J Strohecker AM. Activated ras requires autophagy to maintain oxidative metabolism and tumorigenesis. Genes Dev (2011) 25(5):460–70. doi: 10.1101/gad.2016311 PMC3049287 21317241

[52] Buscail L Bournet B Cordelier P. Role of oncogenic KRAS in the diagnosis, prognosis and treatment of pancreatic cancer. Nat Rev Gastroenterol Hepatol (2020) 17(3):153–68. doi: 10.1038/s41575-019-0245-4 32005945

[53] Levy JMM Towers CG Thorburn A. Targeting autophagy in cancer. Nat Rev Cancers (2017) 17(9):528–42. doi: 10.1038/nrc.2017.53 PMC5975367 28751651

[54] Görgülü K Diakopoulos KN Kaya-Aksoy E Ciecielski KJ Ai J. The role of autophagy in pancreatic cancer: From bench to the dark bedside. Cells (2020) 9(4):1063. doi: 10.3390/cells9041063 PMC7226443 32344698

[55] Di Malta C Siciliano D Calcagni A Monfregola J Punzi S. Transcriptional activation of RagD GTPase controls mTORC1 and promotes cancer growth. Science (2017) 356(6343):1188–92. doi: 10.1126/science.aag2553 PMC5730647 28619945

[56] Yang AN Herter-Sprie G Zhang HK Lin EY Biancur D. Autophagy sustains pancreatic cancer growth through both cell-autonomous and nonautonomous mechanisms. Cancer Discov (2018) 8(3):276–87. doi: 10.1158/2159-8290.cd-17-0952 PMC5835190 29317452

[57] Brown JM Giaccia AJ. The unique physiology of solid tumors: opportunities (and problems) for cancer therapy. Cancer Res (1998) 58(7):1408–16. 9537241

[58] Rausch V Liu L Apel A Rettig T Gladkich J. Autophagy mediates survival of pancreatic tumour-initiating cells in a hypoxic microenvironment. J Pathol (2012) 227(3):325–35. doi: 10.1002/path.3994 22262369

[59] Andersen HB Ialchina R Pedersen SF Czaplinska D. Metabolic reprogramming by driver mutation-tumor microenvironment interplay in pancreatic cancer: new therapeutic targets. Cancer Metastasis Rev (2021) 40(4):1093–114. doi: 10.1007/s10555-021-10004-4 34855109

[60] Chen HT Liu H Mao MJ Tan Y Mo XQ. Crosstalk between autophagy and epithelial-mesenchymal transition and its application in cancer therapy. Mol Cancers (2019) 18(1):101. doi: 10.1186/s12943-019-1030-2 PMC6533683 31126310

[61] Zhu HT Wang DQ Zhang LR Xie XD Wu YY. Upregulation of autophagy by hypoxia-inducible factor-1 alpha promotes EMT and metastatic ability of CD133(+) pancreatic cancer stem-like cells during intermittent hypoxia. Oncol Rep (2014) 32(3):935–42. doi: 10.3892/or.2014.3298 24994549

[62] Wang Y Xiong H Liu D Hill C Ertay A. Autophagy inhibition specifically promotes epithelial-mesenchymal transition and invasion in RAS-mutated cancer cells. Autophagy (2019) 15(5):886–99. doi: 10.1080/15548627.2019.1569912 PMC6517269 30782064

[63] Fuchs Y Steller H. Programmed cell death in animal development and disease. Cell (2011) 147(4):742–58. doi: 10.1016/j.cell.2011.10.033 PMC4511103 22078876

[64] Kang R Tang D Schapiro NE Livesey KM Farkas A. The receptor for advanced glycation end products (RAGE) sustains autophagy and limits apoptosis, promoting pancreatic tumor cell survival. Cell Death Differ (2010) 17(4):666–76. doi: 10.1038/cdd.2009.149 PMC3417122 19834494

[65] Dando I Pacchiana R Pozza ED Cataldo I Bruno S. UCP2 inhibition induces ROS/Akt/mTOR axis: Role of GAPDH nuclear translocation in genipin/everolimus anticancer synergism. Free Radic Biol Med (2017) 113:176–89. doi: 10.1016/j.freeradbiomed.2017.09.022 28962872

[66] Fukui M Kang KS Okada K Zhu BT. EPA, An omega-3 fatty acid, induces apoptosis in human pancreatic cancer cells: Role of ROS accumulation, caspase-8 activation, and autophagy induction. J Cell Biochem (2013) 114(1):192–203. doi: 10.1002/jcb.24354 22903547

[67] Liang JX Ning Z Gao W Ling J Wang AM. Ubiquitin−specific protease 22−induced autophagy is correlated with poor prognosis of pancreatic cancer. Oncol Rep (2014) 32(6):2726–34. doi: 10.3892/or.2014.3508 25241857

[68] Zhang X Schönrogge M Eichberg J Wendt EHU Kumstel S. Blocking autophagy in cancer-associated fibroblasts supports chemotherapy of pancreatic cancer cells. Front Oncol (2018) 8:590. doi: 10.3389/fonc.2018.00590 30568920 PMC6290725

[69] Xie Y Liu J Kang R Tang D. Mitophagy in pancreatic cancer. Front Oncol (2021) 11:616079. doi: 10.3389/fonc.2021.616079 33718171 PMC7953903

[70] Wolpin BM Rubinson DA Wang XX Chan JA Cleary JM. Phase II and pharmacodynamic study of autophagy inhibition using hydroxychloroquine in patients with metastatic pancreatic adenocarcinoma. Oncologist (2014) 19(6):637–8. doi: 10.1634/theoncologist.2014-0086 PMC4041680 24821822

[71] Rebecca VW Nicastri MC McLaughlin N Fennelly C McAfee Q. A unified approach to targeting the lysosome's degradative and growth signaling roles. Cancer Discovery (2017) 7(11):1266–83. doi: 10.1158/2159-8290.cd-17-0741 PMC5833978 28899863

[72] Lan CY Chen SY Kuo CW Lu CC Yen GC. Quercetin facilitates cell death and chemosensitivity through RAGE/PI3K/AKT/mTOR axis in human pancreatic cancer cells. J Food Drug Anal (2019) 27(4):887–96. doi: 10.1016/j.jfda.2019.07.001 PMC9306979 31590760

[73] Wang F Tian X Zhang Z Ma Y Xie X. Demethylzeylasteral (ZST93) inhibits cell growth and enhances cell chemosensitivity to gemcitabine in human pancreatic cancer cells *via* apoptotic and autophagic pathways. Int J Cancers (2018) 142(9):1938–51. doi: 10.1002/ijc.31211 29238973

[74] Zeh HJ Bahary N Boone BA Singhi AD Miller-Ocuin JL. A randomized phase II preoperative study of autophagy inhibition with high-dose hydroxychloroquine and Gemcitabine/Nab-paclitaxel in pancreatic cancer patients. Clin Cancer Res (2020) 26(13):3126–34. doi: 10.1158/1078-0432.ccr-19-4042 PMC8086597 32156749

