# Supplementary material for: The global research and emerging trends in autophagy of pancreatic cancer: A bibliometric and visualized study
Source: Front Oncol. 2022 Oct 3;12:987026. doi: 10.3389/fonc.2022.987026 (PMC9574366; doi:10.3389/fonc.2022.987026)
Supplement: Supplementary file 1 [file Table_1.docx]

((pancreatic cancer) OR (pancreatic carcinoma) OR (pancreatic neoplasm) OR (cancer of pancreas) OR (carcinoma of pancreas) OR (neoplasm of pancreas) OR (pancreatic ductal adenocarcinoma) OR (PDAC)) AND ((autophagy) OR (macroautophagy) OR (microautophagy) OR (chaperone-mediated autophagy) OR (mitophagy) OR (ferritinophagy) OR (aggrephagy) OR (lipophagy) OR (clockophagy) OR (nucleophagy) OR (xenophagy))
