# Supplementary material for: The global research and emerging trends in autophagy of pancreatic cancer: A bibliometric and visualized study
Source: Front Oncol. 2022 Oct 3;12:987026. doi: 10.3389/fonc.2022.987026 (PMC9574366; doi:10.3389/fonc.2022.987026)
Supplement: Supplementary file 2 [file Table_2.docx]

label replace by

peoples r china China

taiwan China

pancreatic carcinoma pancreatic cancer

pancreatic-cancer cells pancreatic cancer

pancreatic adenocarcinoma pancreatic cancer

pancreatic ductal adenocarcinoma pancreatic cancer

pancreatic-cancer pancreatic cancer

pdac pancreatic cancer

cell-death cell death

beclin-1 beclin 1

cancer-cell cancer cells

cancer-cells cancer cells

tumor-cells cancer cells

carcinoma cells cancer cells

tumors cancer

carcinoma cancer
